# The Role of Self-control and Grit in Domains of School Success in Students of Primary and Secondary School

**DOI:** 10.3389/fpsyg.2017.01716

**Published:** 2017-10-11

**Authors:** Xavier Oriol, Rafael Miranda, Juan C. Oyanedel, Javier Torres

**Affiliations:** ^1^Facultad de Educación, Universidad Andres Bello, Santiago de Chile, Chile; ^2^Departamento de Psicología, Universidad Continental, Huancayo, Peru; ^3^Universidad Tecnológica de Chile INACAP, Santiago, Chile

**Keywords:** Self-control, grit, academic self-efficacy, primary and secondary school

## Abstract

**Objective:** Self-control and grit have become two of the most important variables that explain success in different aspects of people's daily life (Duckworth and Gross, 2014). Self-control promotes delayed gratification and directly influences thoughts, emotions, and impulses. On the other hand, grit enhances the achievement of goals through perseverance even before extreme external circumstances. Since both constructs are related, examining them together is compelling, as long as the different nuances that characterize each are taken into account. Two structural equation models (SEM) were conducted to observe the effect of self-control and grit on a more specific indicator of academic success (academic self-efficacy) and a more general indicator of school experience (satisfaction with school).

**Methods:** The first model comprises 5,681 primary students (*M* = 9.05; *SD* = 0.79), and the second 10,017 secondary students (*M* = 14.20; *SD* = 1.04) from Lima, Peru. In both models, the influence of grit and self-control on school satisfaction was observed when taking self-efficacy as a mediator variable.

**Results:** The results show that grit and self-control have strong associations in both primary and secondary students. When estimating the covariance of both constructs, grit is related with academic-self efficacy at both educational stages, but only to satisfaction with school in secondary students. On the contrary, self-control shows a significant relationship with school satisfaction only in primary education. In turn, self-efficacy shows a mediating effect between grit and school satisfaction. After calculating the invariance of the models, differences are observed by gender in the relationships between variables.

**Conclusion:** The results indicate that both constructs are strongly interrelated. Regarding the associations with the indicators of academic success, a need for timely interventions specific to each educational stage is observed.

## Introduction

The most recent academics studies demonstrated that self-control and grit have become two of the most important variables that explain success in different aspects of people's daily life (Duckworth and Gross, 2014). Since both constructs are related, examining them together is compelling, as long as the different nuances that characterize each are taken into account (Duckworth and Gross, 2014; Galla and Duckworth, 2015).

Both self-control and grit have been related to willpower. Specifically, self-control appears to promote delayed gratification and directly influences thoughts, emotions and impulses (Mischel, 2014); while grit is produced by keeping willpower constant and enhances the achievement of goals even before extreme external circumstances (Duckworth et al., 2007; Singh and Jha, 2008).

The experimental studies carried out by Mischel et al. (1989) and Mischel (2014) using the “*marshmallow test*” have been fundamental to understand the concept of self-control at present. These experiments allowed demonstrating the capacity of human beings for delaying gratification; and evidenced the importance of working on willpower from early ages (Mischel, 2014). The research conducted by Baumeister et al. (1998); Baumeister (2014) contributed to this perspective by finding that the human being frequently has to deal with daily life activities that rely heavily on self-control, which may drain willpower. However, it was also observed that positive states of mind induced after these exhausting tasks could relieve such exhaustion (Muraven, 2012).

The daily life of human beings is full on conflicts in which the temptation for immediate desires of higher order needs to be solved or satisfied immediately (Duckworth and Gross, 2014). Therefore, the development of a high capacity of regulation from early ages seems to product a long-term effect, since it promotes the resistance to higher order desires and allows directing behavior toward the achievement of goals (Hofmann et al., 2012). In this sense, the most recent longitudinal studies have also focused on identifying the effects of self-control in the long term, thus concluding that learning to delay gratification during the early ages predicts academic performance in further stages (Duckworth and Carlson, 2013; Duckworth and Gross, 2014; Mischel, 2014). Other studies have underscored that interventions to enhance self-regulation help students to feel less anxiety and tiredness as well as to focus on their studies avoiding external distractors (Oaten and Cheng, 2006; Cranwell et al., 2014). After the onset of adolescence, self-control also enable adolescents who are capable of regulating actions and emotions in the short term to set more long-term goals (Demetriou, 2000; Moilanen, 2007).

In this sense, the development of *grit* is related to more long-lasting goals that sometimes require years of effort to be attained (Duckworth et al., 2007; Duckworth and Gross, 2014). This non-cognitive personality trait is composed of a mixture between consistency in the interests and perseverance, and has been found a predictor of academic success in previous studies (Duckworth et al., 2007; Duckworth and Quinn, 2009), after controlling for educational aspirations and former achievements (Strayhorn, 2013). Nevertheless, a study recently conducted by Ivcevic and Brackett (2014) showed that some indicators of academic success, namely rule violation, behavior recognitions, academic honors, and point average, were predicted by the big five trait “conscientiousness” and the emotion regulation ability, while *grit* did not much contribute to incremental validity.

The above suggests that much more research conducted to demonstrate the effects of grit on different variables of academic success, bearing in mind the changes that take place during the different educational stages. During secondary education, dramatic cognitive, and emotional changes occur, which influence information processing and decision-making (Blakemore and Robbins, 2012; Lerner et al., 2015). Some authors propose that adolescence is a stage of more emotional reactivity, especially due to the changes implied by puberty (Forbes and Dahl, 2010; Forbes et al., 2011). However, during this stage, adolescents also develop the capacity of establishing plans or preparing events that are more distant in time, in contrast to children, whose capacity is more limited to close events (Barkley, 1997). This capacity depends on the functions of the prefrontal cortex, which oversees the orientation to future and experiences, an important maturation process that occurs during adolescence (Blakemore et al., 2007; Burghy et al., 2012).

As for differences by gender in both constructs, the literature on this subject is ambiguous, and thus more studies are needed. Some studies conducted over the last few years have not found differences in self-control between boys and girls (e.g., Jonason and Tost, 2010), whereas others have concluded that men show lower self-control scores than women (Winfree et al., 2006; Weis et al., 2013). These differences may be due to the type of instrument used and the effect of age, which need to be controlled for.

In the case of grit, many studies conducted by Duckworth et al. (2007) did not find differences by gender; however, in other more recent study, women scored higher than men (Christensen and Knezek, 2014).

### Present study

For many researchers, one of the key aspects to explore nowadays is how self-control and grit behave before other variables in the different stages of the life cycle to promote their development (e.g., Vohs and Baumeister, 2004, 2013; Duckworth and Gross, 2014; Mischel, 2014). Overall, there seems to be a significant relationship between both constructs, but people with high levels of self-control are not necessarily gritty and vice versa (Duckworth, 2016).

In this sense, this study intends to observe how more stable patterns such as self-control and grit relate to one another and contribute to different domains of academic success. On the one hand, an outcome variable like self-efficacy is considered an indicator of academic success. This concept refers to the beliefs about the own capacities to learn and successfully perform the academic tasks or a performance in a specific domain (Bandura, 1986, 1997; Høigaard et al., 2015), and has been related to academic engagement, motivation for academic tasks and achievement (Salanova et al., 2011; Oriol-Granado et al., 2017). On the other hand, indicators like school satisfaction have been deemed more global. The concept of school satisfaction alludes to a specific quality of the life domain present in the educational environment, which is defined as the student's evaluation of the positivity of his or her school experiences “as a whole” (Huebner, 2004; Baker and Maupin, 2009; Oriol et al., 2017).

Both grit and self-control are expected to promote self-efficacy for solving academic tasks. However, this variable is also expected to act as a mediator between the patterns of grit and self-control, and as a more global indicator like school satisfaction. Furthermore, it is especially interesting to observe these relationships at ages corresponding to the different educational stages such as primary and secondary education.

To this end, two SEM models are presented, one for primary and other for secondary education, with the purpose of testing the following hypothesis: (1) a strong relationship is expected between self-control and grit in both primary and secondary students (2) Grit and self-control will be predictors of self-efficacy and school satisfaction in students from both educational stages. (3) Academic self-efficacy (which implies efficacy beliefs about specific tasks) would act as a mediator variable between self-control and grit, and as a more global indicator of academic success, like school satisfaction. Finally, (4) differences by gender are expected between the models when comparing the primary and secondary stage.

## Materials and methods

### Participants and procedure

The participants of this study took part in the impact assessment of the project “Escuela Amiga” of Peru's Ministry of Education (MINEDU). The data used for the present article corresponds to the base line of the same. The ethical considerations of the project were approved by MINEDU and endorsed by the ethics committee of the Innovation for Poverty Action (IPA), organization that provided technical assistance to MINEDU in the elaboration of written informed consents for parents and school principals, and written informed assents for students that were verbally explained prior to proceeding data collection. In this sense, the objective of the project was informed prior to the application of the questionnaire and students were informed that they were free not to answer the questionnaire in case they do not want.

In addition, as part of the ethical considerations proposed, IPA and MINEDU created protocols for monitoring data collection. Regarding the application protocol, the Ministry of Education and NGO IPA trained the interviewers on the application of the instrument and the implementation of an emotional containment protocol in case a student was affected by the content of the questionnaire. According to this protocol, in the event that a student experiences a situation affecting his emotional well-being, the survey supervisor should approach to his or her desk and asks her or him how he or she feels. In case of detecting any situation of emotional discomfort, the student is immediately referred to the psychologist or guardian, who is in a space different from the classroom where the survey is applied.

The instrument was applied to 15,825 students in 99 public educational institutions located in Lima Metropolitana. The sample was divided into 10,044 primary students (63.5%) and 5,781 secondary students (36.5%).

The mean age of the primary school sample was 9.05 years (*DE* = 0.81), with men's participation (50.1%) being similar to women's (49.9%). In the case of secondary students, the mean age was 14.2 (*DE* = 1.00) and, just as the primary education students, the distribution according sex was similar between men (49.5%) and women (50.5%).

### Measures

#### Self-control

This scale was based on Moilanen's self-regulatory inventory (Moilanen, 2007). This scale is composed of four items on the short-term regulation dimension, with items related with impulse, attentional and emotional regulation toward immediate objectives, for example “*When I'm sad, I can usually start doing something that will make me feel better,” “After I'm interrupted or distracted, I can easily continue working where I left off.”* The Likert scale used contained five points. The Cronbach's alpha of this sample was 0.60 for secondary and 0.59 primary education.

#### Grit

Based on the GRIT Scale (Duckworth and Quinn, 2009). This scale was adapted to the instrument used for assessing the project “Escuela Amiga,” denominated Single School Well-being Questionnaire (in Spanish, Cuestionario Único de Bienestar Escolar CUBE), and psychometrically validated (MINEDU, 2013, 2014). This scale is composed of eight items, such as “*New ideas and projects distract me from the projects that I already undertook,” “Difficulties (when something takes too long or doesn't work) don't get me down.”* The Likert scale used contained five points. The Cronbach's alpha of this sample was 0.55 for secondary and 0.61 for primary students.

#### Academic self-efficacy

The scale was adapted from the instrument applied by Roeser et al. (1996). This dimension was assessed via three items, for instance, “*If I have enough time, I can do well all my homework.”* The Likert scale used contained five points. The Cronbach's alpha was 0.68 and 0.66 for secondary and primary education, respectively-

#### School satisfaction

This scale was adapted from Long et al. (2012) and translated into Spanish for Peru by Merino Soto (2013). It assesses subjective well-being in school through five items, e.g., “*I learn quite a lot in school,” “In general, I always want to go to school.”* The Likert scale used contained five points. The Cronbach's alpha was 0.85 for secondary education and 0.80 for primary education.

## Results

### Data analysis

Descriptive analysis and correlations were calculated for each dimension by means of the software SPSS v.24. Confirmatory factor analyses, mediations, structural equation models (SEM) and multigroup analysis were computed using the statistical package AMOS 22.0. These analyses were conducted using Maximum Likelihood—since this statistical descriptor has low sensitivity to the non-compliance of the multivariate normality assumption (West et al., 1995)—and Bootstrapping.

To verify the adequate fit of the model, the following robustness indices were used: Comparative Fit Index (CFI) and Tucker-Lewis Index(TLI)—for which values above 0.95 indicate a good fit and, and above 0.90 and acceptable fit (Medsker et al., 1994); Akaike Information Criterion (AIC)—where the lower value indicates the highest parsimony; and the Root Mean Square Error of Approximation (RMSEA)—for which it has been suggested that values below 0.05 are a good fit, while values between 0.05 and 0.08 indicate an acceptable fit (Browne and Cudeck, 1992).

### Descriptive analyses

Demographic characteristics of the sample are shown in Table 1. As well, The descriptive statistics for the analyzed variables present significant differences by educational stage and sex (see Table 2). In the primary stage, the means of the variables exceed those of the secondary stage. Regarding differences by sex, academic self-efficacy and school satisfaction are statistically higher in boys, while self-regulation and grit are statistically higher in girls.

**Table 1:** Demographic characteristics of the sample.

	**Characteristics**		***N***	**%**
Primary	Age			
		7	21	0.2%
		8	1,815	18.1%
		9	6,31	62.8%
		10	1,109	11%
		11	347	3.5%
		12	99	1.0%
		13	59	0.6%
		14	7	0.1%
		Lost	211	2.8%
	Sex			
		Man	4,961	50.1%
		Woman	4,935	49.9%
Secondary	Age			
		12	25	0.4
		13	1266	21.9
		14	2759	47.7
		15	1044	18.1
		16	423	7.3
		17	130	2.2
		18	26	0.4
		Lost	100	1.7
	Sex			
		Man	2,781	49.5%
		Woman	2,842	50.5%

**Table 2:** Descriptive statistics.

	**Total**		**Primary**		**Secondary**		**Men**		**Women**	
	***M***	***DE***	***M***	***DE***	***M***	***DE***	***M***	***DE***	***M***	***DE***
Academic self-efficacy	3.44	0.61	3.56^***^	0.58	3.21^***^	0.60	2.59^***^	0.67	2.62^***^	0.68
Self-regulation	2.82	0.77	2.97^***^	0.78	2.58^***^	0.69	3.41^**^	0.63	3.46^**^	0.60
School satisfaction	3.37	0.67	3.53^***^	0.61	3.08^***^	0.68	2.85^**^	0.75	2.80^**^	0.79
Grit	2.60	0.67	2.82^***^	0.68	2.24^***^	0.51	3.31^**^	0.70	3.43^**^	0.64

** p < 0.01;

*** *p < 0.001*.

The correlations were calculated among all variables at a general level, and by educational stage. As shown in Table 3, all correlations were significant (p < 0.05). Likewise, all of them show a coefficient equal to or above 0.30.

**Table 3:** Correlations between variables at a general level and by educational level.

	**Variable**	**1**	**2**	**3**	**4**
General	1. Grit	–			
	2. Academic self-efficacy	0.44^***^	–		
	3. Self-regulation	0.48^***^	0.44^***^	–	
	4.School satisfaction	0.42^***^	0.56^***^	0.42^***^	–
Primary	1. Grit	–			
	2. Academic self-efficacy	0.41^***^	–		
	3. Self-regulation	0.46^***^	0.43^***^	–	
	4.School satisfaction	0.36^***^	0.53^***^	0.41^***^	–
Secondary	1. Grit	–			
	2. Academic self-efficacy	0.32^***^	–		
	3. Self-regulation	0.35^***^	0.33^***^	–	
	4.School satisfaction	0.30^***^	0.49^***^	0.30^***^	–

*** *p < 0.001*.

### Structural equation modeling analysis

Prior to the calculation of the SEM, the measurement model was calculated at a general level and according to educational stage. The three measurements presented adequate model adjustments, except from the χ^2^/g.l coefficient, whose value was over 5, thus only indicating an acceptable fit (Hu and Bentler, 1999).

In the case of the adjustments of the general measurement model, the adjustment statistics are acceptable (Bentler and Bonett, 1980; Hu and Bentler, 1999; Hair et al., 2007). The assessed indexes are (χ^2^ = 7,105.33, χ^2^/g.l. = 38.82; TLI = 0.90, CFI = 0.91, RMSEA = 0.049). The model adjustment is also adequate for primary students (χ^2^ = 3393.14, χ^2^/g.l. = 18.54; TLI = 0.91, CFI = 0.92, RMSEA = 0.042), and the results of secondary education maintain the same trend (χ^2^ = 3,832.12, χ^2^/g.l. = 20.94; TLI = 0.85, CFI = 0.87, RMSEA = 0.059).

With respect to the factor loading analyzed in the adjustment model, some items of the grit variable have factor loadings below 0.4 in the three models (general, primary y secondary). Therefore, these items were eliminated from the latent variable (García Berumen González et al., 2012), thus forming a new grit variable composed of four items: (1) “*Difficulties (when something takes too long or doesn't work) don't get me down,”* (2) “*I'm persevering, I work hard when I do things,”* (3) “*I always finish what I start”* y (4) “*I'm dedicated and careful.”* In the case of the self-regulation variable, the item “*After I'm interrupted or distracted, I can easily continue working where I left off”* was removed.

After eliminating these items, the indexes of the adjustment model improve at a general level (χ^2^ = 2646.44, χ^2^/g.l. = 27.00; TLI = 0.96, CFI = 0.96, RMSEA = 0.041). The same is true for the primary level (χ^2^ = 1110.88, χ^2^/g.l. = 11.33; TLI = 0.97, CFI = 0.97, RMSEA = 0.032) and secondary level (χ^2^ = 1786.78, χ^2^/g.l. = 18.23; TLI = 0.92, CFI = 0.93, RMSEA = 0.055).

Subsequently, two SEM were conducted according to the educational level of the sample. The proposed model considers self-regulation and grit to be predictor variables of school satisfaction and academic self-efficacy. In turn, academic self-efficacy is a mediator variable between the predictor variables of school satisfaction.

**Figure 1:**
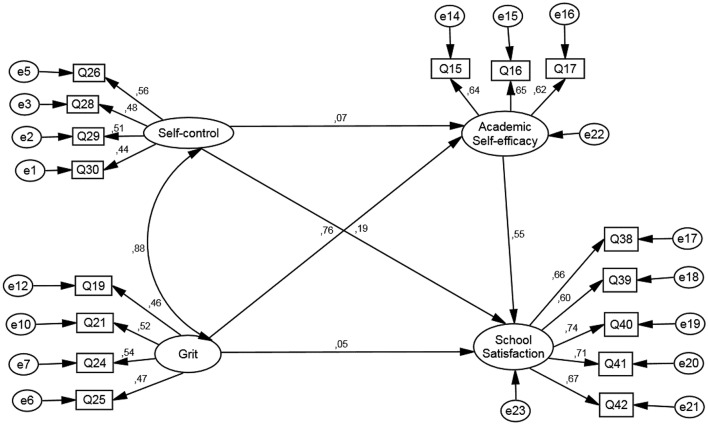
Structural equation modeling for primary students.

The SEM for primary students detailed in Figure 1 shows an adequate fit: χ2/df = 11.34, df = 98; *p* < 0.001; CFI = 0.97; TLI = 0.97; RMSEA = 0.032.

The direct effects are specified in Table 4. The relationship between self-control and academic self-efficacy, together with grit over school satisfaction are not significant (*p* > 0.05). As for the relationship between grit and school satisfaction mediated by academic self-efficacy, there is a total mediation [IC = (0.21, 0.33); *p* < 0.001]. The indirect effect between self-control and school satisfaction through academic self-efficacy turns out to be non-significant [IC = (−0.06, 0.09); *p* > 0.05].

**Table 4:** Indirect effects of the structural equation model for primary students.

**Dependent variable**		**Independent variable**	**Estimator**	**IC**
Academic self-efficacy	<—	Self-control	0.07	[−0.13, 0.22]
Academic self-efficacy	<—	Grit	0.76^***^	[0.61, 0.95]
School satisfaction	<—	Academic self-efficacy	0.55^***^	[0.46, 0.65]
School satisfaction	<—	Grit	0.05	[−0.14, 0.21]
School satisfaction	<—	Self-control	0.19^**^	[0.06, 0.33]

^*^p < 0.05;

** p < 0.01;

*** *p < 0.001*.

*95% confidence interval between brackets*.

**Figure 2:**
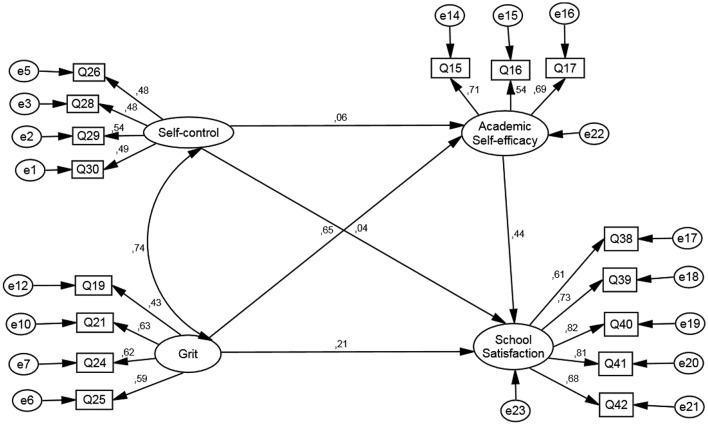
Structural equation model for secondary students.

The SEM for secondary education detailed in Figure 2 shows an adequate adjustment: χ2/df = 18.23, df = 98; *p* < 0.001; CFI = 0.93; TLI = 0.92; RMSEA = 0.055.

Regarding the direct effects of the model, it is observed that the relationship between self-control, and school satisfaction and academic self-efficacy is not significant (*p* > 0.05). The rest of the effects was significant (see Table 5). With respect to the indirect effects, an effect mediated by academic self-efficacy was found between grit and school satisfaction, [IC = (0.21, 0.33); *p* < 0.001]. Lastly, the mediating effect in the relationship between self-control and school satisfaction mediated by academic self-efficacy was not significant [IC = (−0.01, 0.06); *p* > 0.05].

**Table 5:** Indirect effects of the structural equation model for secondary students.

**Dependent variable**		**Independent variable**	**Estimator**	**IC**
Academic self-efficacy	<—	Self-control	0.06	[−0.04, 0.15]
Academic self-efficacy	<—	Grit	0.65^***^	[0.56, 0.74]
School satisfaction	<—	Academic self- efficacy	0.44^***^	[0.38, 0.50]
School satisfaction	<—	Grit	0.21^**^	[0.11, 0.31]
School satisfaction	<—	Self-control	0.04	[−0.03, 0.12]

^*^p < 0.05;

** p < 0.01;

*** *p < 0.001*.

*95% confidence interval between brackets*.

### Invariance analysis

Invariance was calculated by sex for each of the educational stages proposed in the model. The direct effects are presented in Table 6 for each of the educational stages and according sex. In the case of primary education students, the relationship between self-control and school satisfaction is significant for women (*p* < 0.05) and non-significant for men (*p* > 0.05). In the case of secondary education students, the effect of grit on school satisfaction is also significant for women (*p* < 0.05), but not for men (*p* < 0.05).

**Table 6:** Direct effects according sex for primary and secondary students.

			**Man**	**Woman**
**PRIMARY STUDENTS**				
Academic self-efficacy	<—	Self-control	0.86^***^	0.69^***^
Academic self-efficacy	<—	Grit	−0.03	0.15^*^
School satisfaction	<—	Academic self-efficacy	0.60^**^	0.50^**^
School satisfaction	<—	Grit	−0.04	0.06
School satisfaction	<—	Self-control	0.21^**^	0.23^**^
**SECONDARY STUDENTS**				
Academic self-efficacy	<—	Self-control	0.62^***^	0.67^**^
Academic self-efficacy	<—	Grit	0.10	0.03
School satisfaction	<—	Academic self-efficacy	0.51^***^	0.38^**^
School satisfaction	<—	Grit	0.13^*^	0.22^**^
School satisfaction	<—	Self-control	0.08	0.09

* p < 0.05;

** p < 0.01 and

*** *p < 0.001 (bilateral)*.

Table 7 shows significant differences by sex (*p* < 0.05) between the proposed invariance models. In the case of the primary stage, there are no significant differences between the unconstrained model and the measurement weight model, and between the structural weight model and measurement weight model. Nevertheless, significant differences exist between the structural covariance model and the structural weights model. In the secondary stage, significant differences were identified between the unconstrained and the measurement weight model (*p* < 0.05).

**Table 7:** Invariance of models between men and women according to educational stage.

**Models**	**χ^2^**	**df**	**χ^2^/df**	**Δχ^2^**	**Δdf**	**CFI**	**TLI**	**RMSEA**
**PRIMARY**								
Model 1	1,275	196	–	–	–	0.97	0.96	0.02
Model 2	1,286	208	6.18	11	12	0.97	0.97	0.02
Model 3	1,292	213	6.07	6	5	0.97	0.97	0.02
Model 4	1,308	216	6.06	16^**^	3	0.97	0.97	0.02
Model 5	1,344	218	6.17	36^***^	2	0.97	0.97	0.02
Model 6	2,147	234	9.18	803^***^	16	0.95	0.95	0.03
**SECONDARY**								
Model 1	1,783	196	–	–	–	0.94	0.92	0.04
Model 2	1,825	208	8.78	42^***^	12	0.94	0.93	0.04
Model 3	1,840	213	8.64	15^**^	5	0.93	0.93	0.04
Model 4	1,857	216	8.60	17^**^	3	0.93	0.93	0.04
Model 5	1,858	218	8.52	1	2	0.93	0.93	0.04
Model 6	2,128	234	9.09	270^***^	16	0.92	0.92	0.04

** p < 0.01;

*** *p < 0.001. Model 1, Unconstrained; Model 2, Measurement weights; Model 3, Structural weights; Model 4, Structural covariance; Model 5: Structural residuals; Model 6: Measurement residuals*.

## Discussion

First, one of the aspects that arises more interest nowadays is the relationship existing between grit and self-control at the different educational stages. In this sense, we observe high scores between these two constructs in both the correlations and the covariance value. In both self-control and grit, primary students show higher scores than secondary students. These data might seem surprising, since adolescents appear to have an increased maturation of the prefrontal cortex and to be more capable of setting goals and long-term plans (Blakemore et al., 2007). However, in some cases adolescents set goals such as “study to improve their marks” more induced by extrinsic motivation caused by social pressure than by other intrinsic motivation processes (Wong and Csikszentmihalyi, 1991). When this occurs, tedious and sometimes frustrating feelings may arise. During periods of transition to puberty, adolescents have a lot of trouble trying to control their impulses and to make adequate decisions when their objective involves emotional components (Bell and McBride, 2010). When confronted with these frustrating situations, adolescents may behave more impulsively than children and adults, and may feel depressed when not meeting their objectives (Casey and Caudle, 2013). Previous studies show that, during adolescence, intrinsic motivation declines as age increases (Lepper et al., 2005; Corpus et al., 2009) and this could be directly related to the differences in grit and self-control found between children and adolescents. In this sense, developing superordinate goals that compel personal significance and intrinsic motivation in childhood could increase the capacity to manage the innumerable mistakes and frustrations of daily life, especially during adolescence (Duckworth and Gross, 2014).

The second hypothesis proposed that grit and self-control would be predictors of both self-efficacy and school satisfaction at both educational stages. The results show that, calculating the covariance between both constructs, self-control stops relating with the indicators of academic success during adolescents, but it does predict school satisfaction in primary students. These data would be consistent with a recent study conducted by Hofmann et al. (2014), in which it was observed that people with more self-control traits would be happier and more satisfied with their life in general, due to the proactive control of their daily lives. In primary students, grit is related to academic self-efficacy and not with school satisfaction. This construct promotes perseverance in students and might be more useful than self-control to motivational systems. However, it must be noted that perseverance boosted by an extrinsic motivation may generate an obsessive passion than may become maladaptive for the individual in the long term (Vallerand, 2008). In this sense, as previously discussed, it would be better if students experience the type of satisfaction with learning that favors intrinsic motivation mechanisms in these early school stages.

During adolescence, grit does show a significant effect on school satisfaction, albeit with less intensity than academic self-efficacy. At this stage, adolescents orient their lives more to the future, and their cognitive development resembles adults'. This might imply that achieving long-term objectives becomes especially important for adolescence, since these attainments contribute to more global indicators of perceived well-being. Furthermore, studies on well-being report that perseverance in the achievement of objectives promotes a more positive life and constantly high levels of subjective well-being (Huebner, 2004; Hoyle, 2006; Magen and Gross, 2010).

The results of the third hypothesis show a mediating effect between grit and school satisfaction in primary and secondary students, and, on the contrary, the absence of mediation with self-control. As stated above, self-control does not relate to beliefs of academic self-efficacy in the SEM models, and thus no mediating effect is observed. This might be due to the strong effect of the covariance between grit and self-control in both primary and secondary stages.

In the last hypothesis, we expected to find differences by sex between models. In the first place, the descriptive statistics show higher scores for girls in both self-control and grit. These results confirm the findings of some studies where higher scores in girls were observed in both constructs (Weis et al., 2013; Christensen and Knezek, 2014). After calculating invariance between the models, differences are not observed in the unconstrained model for primary students, but they are present in the other three models calculated. With respect to direct effects, it may be seen that grit predicts academic self-efficacy more strongly in boys, while a significant effect of self-control over self-efficacy may be observed only in girls. Girls also show higher scores in the relationship between self-control and school satisfaction. Furthermore, significant differences are found between the models for secondary students; therefore, there is no invariance between them. Girls show higher grit scores for both the relationship with self-efficacy and with school satisfaction. No significant relationships are determined between self-control and the outcome variables.

Finally, it should be considered that one of the limitations of this study is its cross sectional instead of longitudinal nature. It would be very interesting that further research be conducted to follow the evolution of these constructs in the same individuals.

## Conclusion

Grit and self-control are considered stable patterns or traits, yet this does not imply that they cannot be nurtured during the early ages by means of some strategies. Both constructs are strongly related to each other, and also to more specific performance indicators like self-efficacy as well as to more global indicators like school satisfaction. Nevertheless, it is observed that the effect of both variables varies according to age and success indicator. This indicates that specific strategies should be developed to work on: (1) more immediate impulse control; and (2) perseverance and passion for studying more in the long term, with the aim of potentiating intrinsic motivation and self-learning in students (Franco et al., 2016). This should also take into account the massive cognitive and emotional changes that occur during the different educational stages.

## Author contributions

XO: Introduction, data analyses, results, and discussion. RM: Data collection and analyses. JO: Review of the literature and results. JT: Review of the literature and references.

### Conflict of interest statement

The authors declare that the research was conducted in the absence of any commercial or financial relationships that could be construed as a potential conflict of interest. The reviewer MG and handling Editor declared their shared affiliation, and the handling Editor states that the process nevertheless met the standards of a fair and objective review.
